# Current State and Issues of Regenerative Medicine for Rheumatic Diseases

**DOI:** 10.3389/fmed.2022.813952

**Published:** 2022-01-28

**Authors:** Ryusuke Yoshimi, Hideaki Nakajima

**Affiliations:** Department of Stem Cell and Immune Regulation, Yokohama City University Graduate School of Medicine, Yokohama, Japan

**Keywords:** stem cell transplantation (SCT), rheumatic diseases (RDs), therapeutic angiogenesis, regenerative medicine, hematopoietic stem cell (HSC), mesenchymal stem cell (MSC), endothelial progenitor cell (EPC)

## Abstract

The prognosis of rheumatic diseases is generally better than that of malignant diseases. However, some cases with poor prognoses resist conventional therapies and cause irreversible functional and organ damage. In recent years, there has been much research on regenerative medicine, which uses stem cells to restore the function of missing or dysfunctional tissues and organs. The development of regenerative medicine is also being attempted in rheumatic diseases. In diseases such as systemic sclerosis (SSc), systemic lupus erythematosus (SLE), and rheumatoid arthritis, hematopoietic stem cell transplantation has been attempted to correct and reconstruct abnormalities in the immune system. Mesenchymal stem cells (MSCs) have also been tried for the treatment of refractory skin ulcers in SSc using the ability of MSCs to differentiate into vascular endothelial cells and for the treatment of systemic lupus erythematosus SLE using the immunosuppressive effect of MSCs. CD34-positive endothelial progenitor cells (EPCs), which are found in the mononuclear cell fraction of bone marrow and peripheral blood, can differentiate into vascular endothelial cells at the site of ischemia. Therefore, EPCs have been used in research on vascular regeneration therapy for patients with severe lower limb ischemia caused by rheumatic diseases such as SSc. Since the first report of induced pluripotent stem cells (iPSCs) in 2007, research on regenerative medicine using iPSCs has been actively conducted, and their application to rheumatic diseases is expected. However, there are many safety issues and bioethical issues involved in regenerative medicine research, and it is essential to resolve these issues for practical application and spread of regenerative medicine in the future. The environment surrounding regenerative medicine research is changing drastically, and the required expertise is becoming higher. This paper outlines the current status and challenges of regenerative medicine in rheumatic diseases.

## Introduction

Stem cells play an important role in maintaining the structure and function of tissues in our bodies by replenishing old cells with new ones (1). Stem cells have both the ability to divide into cells with the same ability as themselves (self-renewal capacity) and the ability to differentiate into various types of cells (multipotentiality). In recent years, stem cell transplantation (SCT) has been applied to various fields of regenerative medicine by utilizing the properties of stem cells (Table 1).

**Table 1:** Comparison of the properties of cells used in regenerative medicine.

	**ESC**	**iPSC**	**HSC/MSC**	**EPC**
Origin	Embryo	Transgenic somatic cells	Adult tissues	Adult tissues
Differential potential	Pluripotent	Pluripotent	Multipotent	Unipotent
Differentiated cells	All cells	All cells	Limited types of cells	Vascular endothelial cells
Proliferation potential	Very high	Very high	Not high	Low
Allogenic/Autologous	Allogenic	Both	Both	Autologous
Rejection	Possible	•Allogenic: Possible •Autologous: Unlikely	•Allogenic: Possible •Autologous: Impossible	Impossible
Ethical issues	Many	Few	None	None
Clinical issues	Risk of tumorigenesis, Instability of supply	Risk of tumorigenesis, Difficulty in quality control	Nothing particular	Nothing particular
Clinical application status	None	Under clinical trial	Widespread	Under clinical trial

*ESC, embryonic stem cell; iPSC, induced pluripotent stem cell; HSC, hematopoietic stem cell; MSC, mesenchymal stem cell; EPC, endothelial progenitor cell*.

The prognosis of rheumatic diseases is generally better than that of malignant tumors, and they have a chronic course. However, there are some patients with poor prognoses who show resistance to conventional therapy and develop irreversible functional impairment and organ damage. Since the mid-1990's, mainly in Europe and the United States, hematopoietic stem cell transplantation (HSCT) has been tried for such patients with poor prognosis, with the aim of correcting abnormalities of the immune system and reconstructing it (2). HSCT is expected to be widely used in daily clinical practice also in the field of rheumatology.

Mesenchymal stem cells (MSCs) are also attracting attention in regenerative medicine, such as the treatment of intractable skin ulcers in systemic sclerosis (SSc) using the differentiation ability of MSCs into vascular endothelial cells and the treatment of systemic lupus erythematosus (SLE) using the immunosuppressive effect of MSCs (3, 4). Endothelial progenitor cells (EPCs) contained in the mononuclear cell fraction of bone marrow and peripheral blood also can differentiate into vascular endothelial cells (5), and their application to vascular regeneration therapy is being attempted. In addition, induced pluripotent stem cells (iPS cells), which have been recently discovered, are expected to be applied to rheumatic diseases (6). In this paper, we review the current status and challenges of regenerative medicine using stem cells in rheumatic diseases.

## Hematopoietic Stem Cell Transplantation

Among the various types of stem cells, somatic stem cells, which are present in our body, are the closest to practical use in regenerative medicine. In 1957, Thomas et al. performed the world's first transplantation of hematopoietic stem cells (HSCs) from the bone marrow of a healthy donor to a patient with leukemia after anticancer drugs and total-body irradiation (7). Since then, bone marrow transplantation (BMT) has been attempted in many countries around the world, and technological advances through trial and error have greatly improved treatment outcomes. Furthermore, it has been shown that HSCs are present not only in bone marrow but also in peripheral blood and cord blood. Since the late 1980's, allogenic or autologous peripheral blood stem cell transplantation (PBSCT) and allogeneic cord blood transplantation (CBT) have also been applied clinically (8–10). Thus, hematopoietic stem cell transplantation (HSCT) is now a well-established and widespread therapy.

The efficacy of HSCT in correcting and reconstructing immune abnormalities in autoimmune diseases has been previously demonstrated in various animal models of disease (11). In spontaneous autoimmune disease models such as MRL/*lpr* mice, only allogeneic transplantation can suppress the onset of disease and induce remission (12–15), whereas, in antigen-induced autoimmune disease models, both allogeneic and syngeneic transplantation can induce remission (16–18). It is thought that human autoimmune diseases are not caused by genetic predisposition alone but also by environmental factors as well. The result that remission induction was possible even with syngeneic transplantation in the antigen-induced models suggests the possibility of clinical application of autologous PBSCT in autoimmune diseases. In addition, case reports of improvement of autoimmune diseases associated with hematological disorders by HSCT have been accumulated (19).

With this background, the application of HSCT to autoimmune diseases such as SSc, SLE, and rheumatoid arthritis (RA), which are refractory to conventional therapy, has been attempted since the mid-1990's, mainly in Europe and the United States. A total of 3,320 transplant procedures, including 1,634 patients with multiple sclerosis, 652 patients with SSc, 196 patients with Crohn's disease, 168 patients with inflammatory arthritis, and 110 patients with SLE, were registered in the autoimmune disease database of the Blood and Marrow Transplantation (EBMT) from 1994 to 2019 (20). Three thousand one hundred three patients received a first autologous HSCT, and 217 patients received an allogeneic HSCT. The number of procedures per year has increased in recent years, especially in multiple sclerosis and SSc, while other indications such as inflammatory arthritis and SLE have decreased considerably. Analysis of the follow-up period showed a 5-year overall survival rate of 86%, a progression-free survival rate of 49%, a relapse incidence rate of 46%, and non-relapse mortality of 5.3% (21).

Many phase I/II trials have been conducted for SSc (Table 2). The results of HSCT (PBSCT 55 patients, BMT 2 patients) performed on 57 SSc patients enrolled in the registry of EBMT and European League against Rheumatism (EULAR) showed significant improvement in skin sclerosis (22). There are three randomized controlled trials for HSCT in SSc, and most of the data show significant improvement in patient's skin scores and moderate improvement in FVC and DLCO. In an open-label randomized phase II trial (Autologous Non-myeloablative Hematopoietic Stem-cell Transplantation Compared with Pulse Cyclophosphamide Once per Month for SSc; ASSIST), 19 SSc patients with organ involvement received HSCT or 6-month monthly IVCY, Eighty-nine percent of the patients who received IVCY progressed within 1 year of randomization, while none of the patients who received HSCT progressed (23). Seven with confirmed progression on IVCY received an HSCT. Eleven patients who received HSCT and were followed for at least 2 years showed significant improvement in skin score and FVC as compared with baseline. In a European multicenter randomized open-label phase III study comparing autologous PBSCT with intravenous cyclophosphamide (IVCY) in 156 patients with SSc (Autologous Stem Cell Transplantation International Scleroderma; ASTIS), treatment-related mortality at 1 year was higher in the PBSCT group than in the IVCY group (10 vs. 0%), but mortality at 4 years was lower in the PBSCT group (hazard ratio 0.29) (24). In a similar randomized open-label phase II trial of 75 SSc patients in the United States (Scleroderma Cyclophosphamide Or Transplantation; SCOT), treatment-related mortality at 6 years was higher in the PBSCT group than in the IVCY group (6 vs. 0%) while the PBSCT group was superior to the IVCY group in terms of asymptomatic survival (74 vs. 47%) and overall survival (86 vs. 51%), respectively (25). Improvements in screening methods and transplantation techniques have reduced transplanted-related mortality from 10% in the ASTIS trial (24) to 3% in the SCOT trial (25, 26). In a recent EBMT multicenter prospective non-interventional study, higher baseline skin score and older age at transplantation were associated with lower progression-free survival, and CD34+ selection was associated with better response (27). By the accumulation of various evidence, autologous HSCT is now an essential part of the SSc treatment and is supported by the latest recommendations of the EULAR; the recommendations state that HSCT should be considered as a treatment for skin and lung disease in selected patients with rapidly progressive SSc at risk of organ failure (strength of recommendation: (A) (28). Regarding the mechanism by which PBSCT exerts its effects, there are reports that (1) it corrects the Th2 bias of the Th1/Th2 balance in SSc (29), (2) it increases naïve T cells and decreases central memory T cells in peripheral blood *via* thymic reactivation (30), and (3) it maintains self-tolerance by increasing regulatory T cells in peripheral blood (31).

**Table 2:** Clinical trials using regenerative medicine for rheumatic diseases.

**Intervention**	**Comparison**	**Disease**	**Study phase**	**Patient number**	**Treatment effect**	**Treatment toxicity**	**References**
Autologous PBSCT/BMT	-	SSc	I/II	57	Improvement in skin sclerosis at 6–36 months	Treatment-related mortality 8.7%	(22)
Autologous PBSCT	IVCY	SSc	II	19 (10 vs. 9)	Improvement in skin sclerosis and pulmonary function at 12 months (vs. IVCY)	No deaths	(23)
Autologous PBSCT	IVCY	SSc	III	156 (79 vs. 77)	Improvement in skin sclerosis and pulmonary function at 2 years (vs. IVCY), Improvement in event-free survival at 1–10 years (vs. IVCY)	Treatment-related mortality 10.1% at 1 year	(24)
Autologous PBSCT	IVCY	SSc	II	75 (36 vs. 39)	Improvement in the global rank composite score and event-free survival at 54 months (vs. IVCY)	Treatment-related mortality 3% at 54 months and 6% at 72 months	(25)
Autologous PBSCT	-	SLE	I/II	7	Achievement of clinical remission in all cases, Disappearance of serum anti-dsDNA antibody in all cases within 1 month	One death due to invasive central nervous system aspergillosis at 3 months	(32)
Autologous PBSCT	-	SLE	II	50	Decrease in disease activity at 6 months to 5 years, Improvement in pulmonary function at 12–60 months	Treatment-related mortality 2%	(33)
Autologous PBSCT	-	SLE	II	32	Decrease in disease activity at 6 months to 5 years	No treatment-related deaths	(35)
Autologous PBSCT (Unmanipulated cells)	Autologous PBSCT (CD34-selected cells)	RA	II	33 (15 vs. 18)	Decrease in disease activity over a median follow-up of 167 (range 45–374) days (Similar outcomes in both groups)	No deaths	(37)
Autologous PBSCT	-	RA	I/II	14	Decrease in disease activity at 3–12 months	No deaths	(38)
Allogeneic UC-MSCT	-	SLE	I/IIa	16	Decrease in disease activity at 1–24 months	No deaths	(70)
Allogeneic BM-/UC-MSCT	-	SLE	I/II	81	Improvement in renal function and decrease in disease activity at 12 months	No deaths	(71)
Autologous SVF transplantation	-	SSc	I	12	Improvement in hand disability, pain, Raynaud's phenomenon, finger edema and quality of life at 6 months	No deaths	(76)

*PBSCT, peripheral blood stem cell transplantation; BMT, bone marrow transplantation; SSc, systemic sclerosis; IVCY, intravenous cyclophosphamide; SLE, systemic lupus erythematosus; RA, rheumatoid arthritis; UC-MSCT, umbilical cord-derived mesenchymal stem cell transplantation; BM-MSCT, bone marrow-derived mesenchymal stem cell transplantation; SVF, stromal-vascular fraction*.

In SLE, HSCT has been performed for refractory or life-threatening cases of SLE, and several phase I/II trials demonstrated disease remission rates of around 30 to 70% at 5 years and significant improvements in quality of life (Table 2) (32–35). In a single-center prospective study of autologous PBSCT in seven patients with SLE refractory to standard therapy, clinical remission was achieved once in all seven patients, and five patients showed no clinical or serological evidence of SLE activity during a median follow-up of 60 months (32). A single-arm trial of 50 SLE patients refractory to standard therapies and either organ- or life-threatening involvement was conducted, and autologous non-myeloablative PBSCT showed promising results for the SLE Disease Activity Index (SLEDAI) score and serum markers, stabilized nephropathy, and prolonged disease-free survival at 5 years (50%) (33). EBMT registry also showed that autologous PBSCT improved SLEDAI in 28 patients, with an overall survival rate of 81% and disease-free survival rate of 29% at 5 years (34). Autologous PBSCT in patients with SLE refractory to conventional therapy resulted in disease remission of 92% at 1 year and 62% at 5 years after transplantation in 26 patients treated with cyclophosphamide, rabbit ATG, and rituximab as non-myeloablative therapy (35).

In RA, some successful studies of HSCT in RA were reported, but the results were not encouraging considering the cost-benefit balance (Table 2) (36–39). Although progression-free survival and disease-free survival were high in patients with RA compared to other rheumatic diseases, the high relapse rate and the development of biologic agents did not justify the further development of HSCT in RA.

The main advantage of HSCT for rheumatic diseases is the ability to reset the immune system and alter the natural history of the disease by removing T-cell clones involved in the autoimmune response. On the other hand, the main disadvantage of HSCT is the added toxicity to the body from the high doses of chemotherapy and radiation used as part of the conditioning regimen. Future challenges for HSCT in rheumatic diseases include how to reduce transplant-related mortality in terms of the risk-benefit balance of treatment. To this end, improvements should be made in the following areas: optimization of indication criteria, optimization of HSC collection, the necessity of CD34 cell purification, and optimization of pre-transplant procedures.

As for indications for HSCT, the EBMT published its recommendations on indications for HSCT in 2015 (40). This was developed based on prospective clinical trials, registry data, and expert opinion. In patients with SSc, HSCT is indicated for a specific subgroup of patients (those with non-Reynaud's disease for <5 years, modified Rodnan skin score > 15, and major respiratory, cardiac, or renal involvement with documented evidence of onset or clinically significant deterioration within the past 6 months). In patients with SLE, HSCT is optional for certain subgroups of patients (early in the disease course or after at least 6 months of standard therapy with mycophenolate mofetil or cyclophosphamide with persistent or relapsed disease activity as defined by the British Isles Lupus Assessment Group (BILAG) A score with cardiovascular, neurologic, renal, or pulmonary involvement, vasculitis, or autoimmune cytopenia. The American Society for Blood and Marrow Transplantation (ASBMT) has established a multi-stakeholder task force composed of transplant professionals, insurance company representatives, and patient advocates to guide “routine” indications for HCT (41). Recommendation categories of allogeneic HSCT for SSc, SLE, and RA are “Not generally recommended,” while those of autologous HSCT are “Developmental.” The guidelines published by the Japanese Society for Hematopoietic Cell Transplantation also describe the current indication criteria (42). The guideline limits eligible patients to those with life-threatening conditions due to intractable disease, or those whose quality of life is significantly reduced due to sequelae even if the disease itself can be controlled. It also describes the criteria for HSCT indications in each rheumatic disease. The number of institutions with experience in HSCT for rheumatic diseases in Japan is still small, and it is important to accumulate experience in transplantation in the future. It is important to accumulate transplantation experience in the future. For this purpose, it is necessary to strengthen cooperation between rheumatologists and hematologists.

## Mesenchymal Stem Cell Transplantation

Mesenchymal stem cells (MSCs) are somatic stem cells with the ability to differentiate into a variety of tissues and have recently attracted attention as a cell source for regenerative medicine (43, 44) (Table 1). In 1970, Friedenstein et al. (45) revealed the existence of MSCs in guinea pigs as cells with the ability to differentiate into bone. Isolation and culture expansion of human bone marrow MSCs was reported in 1992 (46), and the safety of bone marrow MSC injection into patients was reported as early as 1995 (47). In 1999, Pittenger et al. (48) reported MSCs from human bone marrow as cells with the ability to differentiate into bone, fat, and cartilage. MSCs have been found in various connective tissues such as adipose tissue (49, 50), umbilical cord (51), placenta (52, 53), synovium (54, 55), and dental tissues (56, 57) in addition to bone marrow (46, 48), and are thought to play an important role in repair and homeostasis in each tissue. MSCs can differentiate not only into mesenchymal cells such as bone, cartilage, and adipocytes but also into tissue cells of ectodermal origin such as neurons and endodermal origin such as hepatocytes (58–61).

Clinical trials of regenerative medicine using MSCs have been actively conducted around the world, and the U.S. National Library of Medicine's clinical research database “ClinicalTrials.gov” has registered about 1,300 clinical trials as of November 2021. In 2011, Hearticellgram®-AMI, autologous bone marrow-derived MSCs (BM-MSCs), was approved in Korea as the world's first stem cell therapy for acute myocardial infarction (62). Subsequently, several stem cell drugs, such as MSC-rich cryopreserved placental membrane for the treatment of diabetic foot ulcers (63) and allogeneic BM-MSCs for the treatment of acute GVHD utilizing their immunosuppressive function (64, 65), have also been developed and marketed. Recently, Japan has conditionally approved an autologous BM-MSC drug for the treatment of spinal-cord injury although some researchers believe that this approval is premature (66).

In 2008, the first case of BM-MSC transplantation for the treatment of rheumatic diseases was reported by a German group (67). The patient had severe progressive diffuse cutaneous SSc seropositive for anti-Scl-70 antibodies, and digital ulcers and skin sclerosis improved after intravenous injection of allogeneic BM-MSCs. Later, the same group reported that skin sclerosis and limb ulcers improved in 4 of 5 SSc patients who underwent allogeneic BM-MSC transplantation, and no serious adverse events were observed (68). In addition, there is a report that autologous BM-MSC transplantation in SSc patients with gangrene improved blood flow in the extremities on angiography, and areas of necrotic skin were reduced (69).

MSC transplantation has been attempted in SLE patients because of the immunosuppressive effect of MSCs (Table 2). A Chinese group reported that umbilical cord-derived MSC (UC-MSC) transplantation in 16 patients with SLE refractory to standard therapy resulted in significant improvement in SLEDAI and renal function at a median observation period of 8 months (70). Another report by the same group showed that 81 patients with Class III, IV, or V lupus nephritis refractory to immunosuppressive therapy received intravenous allogeneic BM- or UC-MSCs, and about 60% of the patients achieved renal remission after 12 months (71).

In the past, bone marrow was mainly used as a source of MSCs. However, adipose-derived MSCs (ASCs) have been widely used in regenerative medicine because adipose tissue contains a large amount of MSCs and they can be easily obtained from subcutaneous adipose tissue by liposuction (72). Because ASCs can differentiate into vascular endothelial cells and improve blood circulation in a mouse model of hindlimb ischemia (73), ASC transplantation for critical limb ischemia has been tried and the data demonstrated the feasibility and safety (74). In SSc, an Italian group reported that subcutaneous transplantation of autologous ASCs and hyaluronic acid in 6 cases resulted in improvement of skin sclerosis in all cases at 1 year with no adverse events (75). In addition, a French group subcutaneously injected stromal-vascular fraction (SVF) containing autologous ASCs in 12 patients with SSc and found that skin thickness, finger edema, Raynaud's phenomenon, hand disability, pain, and quality of life improved at 6 months after administration without any severe adverse events (76).

MSCs are expected to be effective in regenerative medicine in a wide range of fields including rheumatic diseases, and various clinical studies have been conducted. However, most of these clinical studies have shown short-term improvement in clinical symptoms, and the number of cases is still small. In the future, it is necessary to investigate the long-term clinical effects of MSCs in larger-scale clinical studies to establish the evidence. In addition, the standardization and optimization of technologies for regenerative medicine using MSCs will also be an issue in the future.

## Regenerative Medicine Using Induced Pluripotent Stem Cells

Pluripotent stem cells are stem cells that can differentiate into any type of cells, unlike somatic stem cells, and include embryonic stem cells (ESCs) and induced pluripotent stem cells (iPSCs). In 1981, two independent groups, Evans et al. and Martin et al., established ESCs in mice (77, 78). In 1998, Thomson et al. (79) established the world's first human ESCs. The problem of immune rejection has been a barrier to the application of ESCs in regenerative medicine (Table 1). More importantly, bioethical issues have always been a concern in research and medical treatment using ESCs as these cells are generated from fertilized eggs (80).

In 2006, Takahashi et al. (6, 81) established the world's first iPSCs from mouse fibroblasts, and iPSCs were also established from human fibroblasts in 2007. iPSCs are generated from mature somatic cells and do not pose the problems of both rejection response and bioethics as pointed out for ESCs (Table 1), so research on regenerative medicine using iPSCs has rapidly expanded in recent years. In 2014, a research group led by RIKEN and others conducted the world's first iPSC-based regenerative therapy for exudative age-related macular degeneration, in which retinal pigment epithelium derived from the patient's own iPSCs was transplanted under the retina, and confirmed its long-term efficacy (82, 83). Subsequently, a case was reported in which a patient with idiopathic Parkinson's disease was transplanted with midbrain dopaminergic progenitor cells differentiated *in vitro* from autologous iPSCs, and Parkinson's symptoms improved (84). Now clinical trials of regenerative medicine using autologous iPSCs for various disorders, such as thrombocytopenia, recessive epidermolysis bullosa dystrophica, and muscular dystrophy are also undergoing or planned.

iPSCs are expected to be the starting point for personalized medicine with autologous cell therapy. In contrast to allogeneic cell therapies, autologous iPSCs therapies can treat a variety of diseases without the need for immunosuppression. Autologous iPSCs therapies can be used to treat all patients, not just those not covered by HLA haplobanks. At present, there is no information on the development of regenerative medicine using iPSCs in rheumatic diseases. Ikuno et al. (85) at Kyoto University have developed a technology to efficiently generate vascular endothelial cells from human pluripotent stem cells, which is expected to be applied to vascular regeneration therapy for limb ischemia associated with rheumatic diseases such as SSc. Basic research on the regeneration of cartilage using iPSCs is in progress and may be applied to patients with rheumatoid arthritis whose cartilage is damaged (86, 87).

In human trials involving transplantation of autologous iPSC-derived cells, the longest follow-up has been reported to be 4 years after transplantation, and so far, no serious side effects have been reported, although the number is limited (82–84). Nonetheless, there are still many issues to be solved in regenerative medicine using iPSCs. The formation of teratomas due to the contamination of undifferentiated iPSCs, the possibility of cancerization due to genetic damage during the process of iPSC generation and cultivation, and the development of technology to obtain a sufficient amount and quality of cells for transplantation and a tissue structure suitable for transplantation are major issues for the future. As it takes several months to reprogram and differentiate somatic cells harvested from a patient to produce a cell therapy for the patient (88), optimizing this manufacturing timeline would help ensure that patients receive iPSC-based therapies in a timely manner.

## Therapeutic Angiogenesis Using Vascular Endothelial Progenitor Cells

In 1997, it was discovered that a fraction of adult peripheral blood mononuclear cells (PBMCs) differentiate into vascular endothelial cells in culture and was named endothelial progenitor cells (EPCs) (5). EPCs are positive for the surface antigen CD34 and are a distinct population from MSCs, which are negative for CD34. From the standpoint of embryology and histology, neovascularization can be divided into two types: (1) vasculogenesis, in which hematopoietic stem cells (HSCs) and vascular endothelial cells differentiate from blood islands composed of hemangioblasts in early embryonic stages to form primitive blood vessels, and (2) angiogenesis, in which existing vascular endothelial cells undergo sprouting and migration (89). In the past, it was thought that angiogenesis in adults depended solely on angiogenesis. However, it was revealed that EPCs are present in the circulating blood of adults and are involved in the development of new blood vessels, suggesting that vasculogenesis, which was thought to exist only in the fetal period, may also be established in the adult (5). Subsequent studies revealed that EPCs originate from the bone marrow and are mobilized into the peripheral blood and incorporated into angiogenic sites as needed (90, 91).

From these cellular characteristics, basic research on vascular regeneration therapy was conducted using an animal model of lower limb ischemia. Intravenous administration of cultured human peripheral blood EPCs to immunodeficient mice model of lower limb ischemia improved blood flow in the limb due to angiogenesis (92). In addition, transplantation of autologous bone marrow mononuclear cell (BM-MNC) fractions, which contain EPCs, into the ischemic limb of a rabbit model of lower limb ischemia resulted in angiogenesis and improved blood flow in the limb (93), demonstrating the efficacy of vascular regeneration therapy using BM-MNCs or EPCs.

Based on the results of the basic research described above, a multi-center clinical study of Therapeutic Angiogenesis by Cell Transplantation (TACT) was conducted, in which bone marrow was harvested from the iliac bone of patients with critical limb ischemia and BM-MNC fractions containing EPCs were isolated and transplanted into the ischemic site. When patients with bilateral lower limb ischemia were randomly injected with BM-MNCs in one lower limb and PBMCs in the other limb as a control, the ankle-brachial index, transcutaneous oxygen pressure, rest pain, and pain-free walking time of the limb infused with BM-MNCs improved significantly at 4 weeks as compared to the limb infused with PBMCs (94). In a long-term efficacy and safety study, patients with arteriosclerosis obliterans or thromboangiitis obliterans were implanted with BM-MNCs and maintained significant improvements in leg pain scale, ulcer size, and pain-free walking distance for at least 2 years after treatment (95–97). In the group of patients who responded to the treatment, the number of circulating CD34^+^ and CD133^+^ cells increased continuously for 1 month after treatment, while the number did not increase in the non-responder group (98).

It has been reported that the absolute number of circulating EPCs and their ability to differentiate into vascular endothelial cells are reduced in patients with SSc (99). Thus the therapeutic angiogenesis using autologous BM-MNCs was applied to eight SSc patients with refractory skin ulcers and found that all ulcers disappeared within 6 months, and the improvement of pain was also excellent (100). As the results of therapeutic angiogenesis using autologous BM-MNCs in 69 patients (39 SSc, 30 other collagen diseases) with severe ischemic limb caused by collagen disease accumulated in the TACT study, the 10-year overall survival rate, major amputation-free rate, and amputation-free survival rate were 67.6, 90.9, and 61.2%, respectively with the adverse events occurred in 8.7% (101). The 10-year major amputation-free rates were 97.4% in the SSc group and 82.6% in the other collagen disease group, and the rate of limb salvage tended particularly to be higher in the SSc group among the collagen diseases (102).

The efficacy of transplantation of mononuclear cell fractions containing EPCs in peripheral blood mobilized from bone marrow by subcutaneous injection of G-CSF into the ischemic region is also currently being investigated. Since smoking, aging, and underlying diseases such as diabetes and dyslipidemia have been shown to reduce the quantity and quality of EPCs, the search for ways to improve EPC function will be an important issue in improving transplantation outcomes in the future.

## Discussion

In this paper, we have described the current state and issues of regenerative medicine in rheumatic diseases. Regenerative medicine is attracting so much attention that it is fundamentally changing the paradigm of treatment. It has the potential to become a new treatment option for patients with rheumatic diseases with poor prognoses. Especially in SSc, there is currently no effective drug therapy to prevent tissue fibrosis and restore lung function, so the application of regenerative medicine is strongly expected.

On the other hand, regenerative medicine still has issues to be solved not only in terms of technology but also in terms of society. Regenerative medicine is being actively debated in many countries, including its efficacy and ethical issues. Up to now, there has been a miscellaneous mixture of various regenerative medicine, from clinical research to medical treatment. Because regenerative medicine research is conducted in parallel with patient care, the various groups affected by the care inevitably participate in the debate. In addition to discussion based on avoiding harm, providing benefits, and respecting individual autonomy and justice, problems arise due to the lack of legal regulation of stem cell research and practice. The environment surrounding regenerative medicine research is changing rapidly, and the amount of expertise required is increasing daily. To fully ensure the rights and safety of subjects in clinical trials in the field of regenerative medicine, it is necessary to have clinical research coordinators who have expertise in scientific, ethical, and legal fields. How to realize regenerative medicine while cooperating with society will be an important issue essential for the future development of regenerative medicine.

## Author Contributions

RY conceived the review project and devised the manuscript. HN critically discussed the review. All the above-listed authors edited the manuscript.

## Funding

This work was supported by JSPS KAKENHI Grant Number JP19K08914 (RY), AMED Grant Number JP21bk0104107 (RY), and the SENSHIN Medical Research Foundation (RY).

## Conflict of Interest

The authors declare that the research was conducted in the absence of any commercial or financial relationships that could be construed as a potential conflict of interest.

## Publisher's Note

All claims expressed in this article are solely those of the authors and do not necessarily represent those of their affiliated organizations, or those of the publisher, the editors and the reviewers. Any product that may be evaluated in this article, or claim that may be made by its manufacturer, is not guaranteed or endorsed by the publisher.
